# Exploring the Link Between Motor Functions and the Relative Use of the More Affected Arm in Adults with Cerebral Palsy

**DOI:** 10.3390/s25030660

**Published:** 2025-01-23

**Authors:** Isabelle Poitras, Jade Clouâtre, Alexandre Campeau-Lecours, Catherine Mercier

**Affiliations:** 1 Centre for Interdisciplinary Research in Rehabilitation and Social Integration, CIUSSS de la Capitale-Nationale, Quebec City, QC G1M 2S8, Canada; isabelle.poitras@ucalgary.ca (I.P.); jade.clouatre.1@ulaval.ca (J.C.); alexandre.campeau-lecours@gmc.ulaval.ca (A.C.-L.); 2 School of Rehabilitation Sciences, Laval University, Quebec City, QC G1V 0A6, Canada; 3 Department of Mechanical Engineering, Laval University, Quebec City, QC G1V 0A6, Canada

**Keywords:** accelerometry, activities of daily living, motor function, cerebral palsy, bimanual coordination, upper extremity

## Abstract

Individuals with hemiparetic cerebral palsy (CP) exhibit reduced use of their more affected (MA) arm, yet the factors that influence its use during activities of daily living remain elusive. The objectives of this study were to describe the relative use of the MA arm during an ecological task, examine its relationship with the level of impairment, and investigate its association with performance in various unilateral and bilateral tasks. Methods: Participants took part in two sessions comprising robotic assessments and clinical assessments of motor functions, as well as accelerometry measurement during kitchen tasks. Four variables were derived from accelerometry data. Stepwise regression analyses were used to identify the best contributors to the accelerometry variables among robotic and clinical assessments. Results: Nineteen adults with CP (34.3 years old ± 11.5; MACS I = 7, II = 6, III = 6) were included. The *Use Ratio* measured during the kitchen tasks ranged between 0.10 and 0.63. The best predictors of all accelerometry metrics were two bilateral assessments (r^2^ = 0.23–0.64). Conclusions: The importance of assessing bilateral tasks was reaffirmed by the key role played by two bilateral tasks in determining the relative use of the MA arm. The results support the use of intensity-based accelerometry metrics to measure MA arm use.

## 1. Introduction

Individuals who have hemiparetic cerebral palsy (CP) exhibit more pronounced motor impairments in one hand than the other [1]. The arm displaying more impairments is referred to as the more affected (MA) arm, while the other arm is referred to as the less affected (LA) arm. This disparity in motor function between the two arms leads to increased attention given to the MA arm, for both assessments and interventions [2]. Indeed, most assessments that are used to characterize upper extremity motor functions are either unilateral—extrapolating impairments observed in the MA arm to bilateral motor functions —or subjective—relying on self-reported questionnaires (e.g., ABILHAND-Kids [3]) or clinicians’ observations (i.e., Assisting Hand Assessment [4]). While these assessments provide valuable insights into the performance of the MA arm, they are subject to certain biases (e.g., underestimation of bilateral impairment, recall bias, reliance on the clinician’s experience, etc.) [5,6,7]. Furthermore, bilateral upper extremity motor functions have been underexplored in adults with CP [8,9], leading to even fewer validated options for quantitative clinical assessments in this population, while the assessments that are available do not offer normative data [7,10]. This is an important concern, as most activities of daily living require the use of both arms in a coordinated manner.

Interestingly, the use of robotic devices for quantifying upper extremity impairment has extended to various neurologically impaired populations, including individuals who have experienced stroke [11], people living with multiple sclerosis [12], children with CP [13] and, more recently, adults with CP [14]. This technology enables quantitative assessment of both bilateral and unilateral upper extremity functions, providing normative values that allow for comparison with age- and sex-matched healthy controls [15]. This quantitative approach allows for the use of standardized tasks to tackle the mechanisms that underlie the observed decrease in motor performance. This system has been used to assess performance in different types of bilateral tasks, which can be classified according to a model [16] in children [17] and adults [14,18] with CP. This model classifies bilateral tasks according to two characteristics: (1) the symmetry of arm movements (asymmetric or symmetric task); and (2) the conceptualization of the task goal (independent goals or common goal). Children with CP used different motor strategies compared with their peers when performing symmetric and asymmetric tasks while also demonstrating worse overall motor performance. This suggests that the type of task has a significant impact on their motor performance [19,20]. However, little is known about the bilateral performance of adults with cerebral palsy during symmetric and asymmetric tasks. Indeed, only three studies have addressed this question: one identified worse performance during an asymmetric task [14], while the other two showed that adults with CP tend to slow down their less affected arm to match the capabilities of their more affected arm [8,9]. The use of robotic devices gives interesting insights on potential reasons explaining bilateral impairments. However, a primary limitation of robotic devices is that they are not ecological because they restrict the number of degrees of freedom and the influence of gravity. This potentially limits the ability to generalize conclusions to a broader context.

Recently, the use of accelerometers has been proposed to address this limitation and offer a more ecologically valid assessment of upper extremity use during daily activities. These systems are portable, small, easy to use, and capable of providing valid results across various populations [21,22,23], including in adults with CP [24,25]. Accelerometers generally quantify either the intensity of arm use [21,26] or the amount of time of arm use [27,28]. Measuring arm use in the context of daily activities is particularly important given a phenomenon known as “developmental disregard” in individuals with CP. Developmental disregard is defined as the underuse of the MA arm during activities of daily living, despite assessments of the upper extremity’s actual capacity indicating that the person should be capable of performing the task with their MA arm or both arms [29,30,31,32]. This underuse could lead to a decrease in motor capacity in the unused hand, resulting in further disabilities [29,32]. However, accelerometry data do not provide information on the reason why the MA arm is not used to a greater extent. As a result, measuring developmental disregard and understanding which factor contributes to it remains a challenge.

Given the fact that each assessment method has strengths and limitations, a multimodal assessment approach was used, combining clinical, robotic, and accelerometry assessments as the potential to provide insights on the relationship between upper extremity capacities and upper extremity use in real-world contexts. Therefore, the first objective of this study was to describe the relative use of the MA arm during an ecological task and to assess the difference of relative use of the MA across different levels of motor impairments (Manual Ability Classification System (MACS) I, II, and III) in adults with CP. The second objective was to assess the relationship between the relative use of their MA arm during an ecological task and their motor functions, measured using either clinical or robotic assessment tools.

To attain these two objectives, we assessed MA arm use using accelerometers worn on each wrist during kitchen tasks. The kitchen task was chosen as it is a common everyday activity requiring numerous bilateral movements. Four metrics were derived, focusing either on the intensity or duration of arm use, as well as on either the global relative use of each limb or specifically on periods of time where both arms were used in combination one with the other. We also employed a set of clinical and robotic assessments to measure both bilateral and unilateral motor functions. Because clinical and robotic assessments have very different requirements (primarily given that clinical assessments have a strong focus on manual functions while robotic ones focus on proximal motor control), both types of metrics were tested in separate regression models.

## 2. Materials and Methods

### 2.1. Participants

Participants were recruited from various sources, including through health records at the Centre Intégré Universitaire de Santé et de Services Sociaux de la Capitale-Nationale (CIUSSS-CN), through patient organizations, and through the Université Laval mailing list. Eligible participants met specific criteria, including being aged 18 to 65 years old, having a diagnosis of hemiparetic CP, being capable of performing a transfer with minor assistance to sit in the robotic device, and having a level of I, II, or III (mild to moderate impairments) on the MACS scale. Exclusion criteria included cognitive impairments or uncorrected visual problems that could interfere with the assessment tasks. Ethical approval for the study was obtained from the local ethics committee (Ethics #2018-609, CIUSSS-CN), and all participants provided written informed consent before participation.

The MACS level is a clinical assessment used to characterize participants’ ability to manipulate objects during everyday activities. Level I indicates mild, barely visible impairments, while level III indicates significant difficulties in manipulating objects, requiring task adaptation.

### 2.2. Experimental Setup

**Figure 1:**
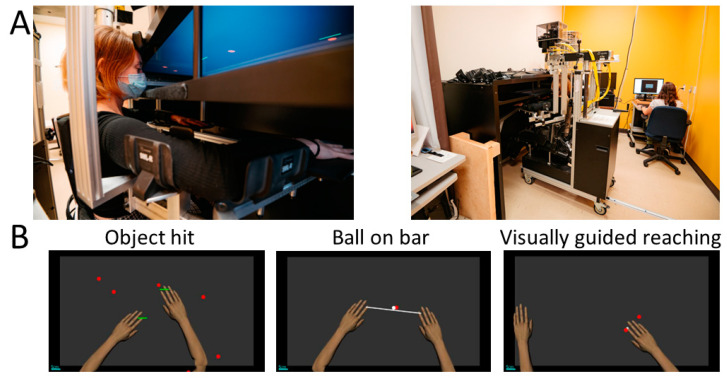
(**A**) Experimental setup for the Kinarm Exoskeleton Lab and (**B**) workspace representation of the Object Hit, Ball on Bar, and Visually Guided Reaching tasks.

**Figure 2:**
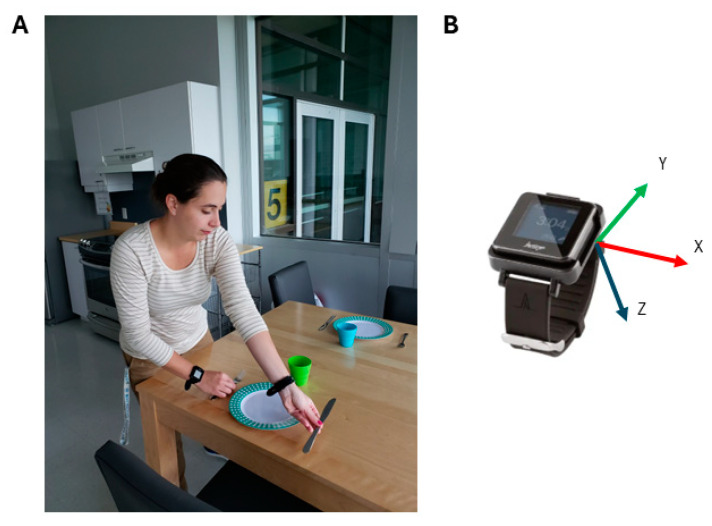
Experimental setup for the accelerometry assessment. (**A**). Participant wearing one accelerometer at each wrist while setting the table. (**B**). Accelerometer used for the assessment including a representation of the *x*-, *y*-, and *z*-axes.

Participants engaged in two assessment sessions, each lasting approximately three hours. The procedure for each session is described in Table 1. The session duration allowed time for participants to take a break between each assessment, reducing the potential impact of fatigue on motor performance. These sessions included various robotic tasks and clinical assessments. The first session involved performing robotic tasks using a bilateral Kinarm Exoskeleton Lab (Kinarm, Kingston, ON, Canada) (see Figure 1 for a visual representation of the experimental setup), along with four of the clinical assessments. The second session involved performing an Observation-based assessment of the involvement of the MA arm as well as a set of kitchen tasks. Each participant chose the recipe of their choice (the choices were baking a cake, baking cookies, or making marshmallow treats). To complete the session, participants were required to gather ingredients from the pantry and bring them to the kitchen, prepare the recipe, clean up, set the table, prepare tea or coffee, eat the dessert, drink the tea or coffee, clear the table, and wash the dishes (duration of the task between 1 h 30 min and 2 h 30 min). The duration of the task varied across participants, as the more impaired individuals generally took longer to complete the task compared with those with milder impairments. However, all participants completed the task, and the analyses were selected to minimize the impact of this difference on the accelerometry results (i.e., relative metrics). The kitchen tasks required participants to move around the kitchen, either by walking or by using a wheelchair. They wore an ActiGraph GT9X Link (sampling rate of 100 Hz, internal memory of 4 GB, accelerometer dynamic range of ±8 g, ActiGraph LLC, Pensacola, FL, USA) on each wrist while performing the task (i.e., accelerometry metrics were calculated from this task; see Figure 2 for the experimental setup for the accelerometry assessment). The two sessions were scheduled less than two weeks apart for most participants, except for one participant who experienced a four-week gap due to being affected by COVID during this period. The robotic tasks included three motor tasks, one bilateral asymmetric independent goal task (Object Hit), one bilateral symmetric common goal task (Ball on Bar), and one unilateral task (Visually Guided Reaching). The clinical assessments included five motor tasks, one bilateral asymmetric independent goal task (Observation-based assessment of the involvement of the MA arm), one bilateral symmetric common goal task (Two-Arm Coordination Test), two unilateral tasks (Jebsen–Taylor Hand Function Test and Box and Block test), and one measurement of grip strength (Jamar hand-grip dynamometer). The tasks were selected to complement each other in terms of type (i.e., there was a combination of bilateral and unilateral tasks).

### 2.3. Accelerometry Processing

The extraction of raw accelerometry data was conducted using ActiLife 6 software (ActiGraph LLC, Pensacola, FL, USA, sampling rate of 100 Hz). The offline pre-processing of data was performed using a custom MATLAB program (MATLAB version 9.6.0 (R2022b), MathWorks Inc., Natick, MA, USA) as detailed in [33], resulting in the calculation of activity counts (ACs). ACs were computed using the vector magnitude (AC = $\sqrt{\;{s_{xi}}^{2}+{s_{yi}}^{2}+{s_{zi}}^{2}}$), representing the norm of the AC on the *x*-, *y*-, and *z*-axes ($s_{xi}$, $s_{yi}$, and $s_{zi}$ denote the sum of AC for a 1 s epoch). The data were then separated into epochs of 1 s, as demonstrated as optimal in adults with CP [25]. Accelerometry data were only gathered during the kitchen task.

### 2.4. Metrics Calculations

Four metrics (detailed below) were computed to measure the relative use of each limb: 1—*Magnitude Ratio*; 2—*Use Ratio*; 3—*Percentage of Bilateral Use*; 4—*Bilateral Arm Use Index* (*BAUI*). For the *Use Ratio* and the *Percentage of Bilateral Use*, an AC threshold of 100 was employed as these are time-based measurements, and this method has been validated by [24]. These four accelerometry metrics made it possible to test the combination of two factors: 1—focusing either on the intensity (i.e., *Magnitude Ratio* and *BAUI*) or duration (i.e., *Use Ratio* and *Percentage of Bilateral Use)* of arm use; and 2—focusing either on the global relative use of each limb (i.e., *Magnitude Ratio* and *Use Ratio*) or specifically on periods of time when both arms are used in combination with one another (i.e., *BAUI* and *Percentage of Bilateral Use*).

A fifth metric, *Bilateral Magnitude*, was extracted to provide visual presentation of the data, but it was not used for analyses.

#### 2.4.1. *Magnitude Ratio* (Metric Focusing on Intensity + Global Relative Use)

The *Magnitude Ratio* represents the contribution of each upper extremity in a specific activity [26]. A vector of *Magnitude Ratio* was calculated for each epoch of 1 s, allowing us to visualize data for the whole task. The formula used was as follows:

(1) $$Magnitude\;Ratio=\log\frac{AC\;of\;the\;MA\;arm}{AC\;of\;the\;LA\;arm}$$

where the AC of the MA arm represents the total AC of the MA arm, while the AC of the LA arm represents the total AC of the LA arm.

This yielded a value between −7 and 7 for each epoch of 1 s. A value of −7 represented more use of the LA arm, while a value of 7 represented more use of the MA arm. Movements that were only unilateral (e.g., where the LA arm was moving while the MA arm was still) were given an artificial value of −7 for the LA arm and 7 for the MA arm, as presented in [26]. A value of 0 indicated equal use of both arms. To gain an overview of the overall use of the upper extremities, the median value of the vector was calculated.

#### 2.4.2. *BAUI* (Metric Focusing on Intensity + Bilateral Use)

The *BAUI* represents the ratio of the intensity of bilateral use [21]. (*BAUI* > 1 represents a higher intensity of use of the MA arm, *BAUI* < 1 represents higher intensity of use of the LA arm, *BAUI* = 1 represents equal intensity of u of both arms). The formula used was as follows:

(2) $$BAUI=\frac{\sum ({AC}_{MA}\;when\;{AC}_{MA}\neq 0\;and\;{AC}_{LA}\neq 0)}{\sum ({AC}_{LA}\;when\;{AC}_{MA}\neq 0\;and\;{AC}_{LA}\neq 0}$$

where $\sum ({AC}_{MA}\;\neq 0\;AND\;{AC}_{LA}\neq 0)$ represents the summation of AC where both arms (MA and LA) are moving simultaneously.

#### 2.4.3. *Use Ratio* (Metric Focusing on Duration + Global Relative Use)

The *Use Ratio* represents the proportion of time one arm was used in relation to the other (*Use Ratio* > 1 represents more use of the MA arm, *Use Ratio* < 1 represents more use of the LA arm, *Use Ratio* = 1 represents equal use of both arms). The formula used was as follows:

(3) $$Use\;Ratio=\frac{movement\;duration\;LA\;arm\;({AC}_{LA}\geq 100)}{movement\;duration\;MA\;arm\;({AC}_{MA}\geq 100)}$$

where the ${AC}_{LA}\geq 100$ represents the AC of the LA arm exceeding the threshold of 100, and ${AC}_{MA}\geq 100$ represents the AC of the MA arm exceeding the threshold of 100.

#### 2.4.4. *Percentage of Bilateral Use* (Metric Focusing on Duration + Bilateral Use)

The *Percentage of Bilateral Use* represents the percentage of time the two arms moved simultaneously. The formula used was as follows:

(4) $$Percentage\;of\;Bilateral\;Use=\frac{\sum ({AC}_{MA}\;\geq 100\;AND\;{AC}_{LA}\geq 100)}{\sum {AC}_{total}}$$

where ${AC}_{MA}\;\geq 100\;AND\;{AC}_{LA}\;\geq 100$ represents the number of epochs where the AC of the MA arm and the AC of the LA arm exceeded the threshold of 100, and the ${AC}_{total}$ represents the number of epochs where at least one arm was moving.

#### 2.4.5. *Bilateral Magnitude*

As mentioned above, the *Bilateral Magnitude* was extracted solely for descriptive purposes. In combination with the *Magnitude Ratio*, it makes it possible to provide a visual representation of the intensity of the use of upper extremities use by means of density plots [26].

A vector of *Bilateral Magnitude* was calculated for each epoch, allowing us to visualize data for the whole task. The formula used was as follows:

(5) Bilateral Magnitude=AC of the MA arm+AC of the LA arm

where the AC of the MA arm represents the total AC of the MA arm, while the AC of the LA arm represents the total AC of the LA arm.

### 2.5. Robotic Assessments

For all robotic assessments, the raw values of the *Task Score* were converted to a z-score by the system based on a methodology presented here: https://kinarm.com/kinarm-products/kinarm-standard-tests (accessed on 20 January 2025).

#### 2.5.1. Object Hit

In the Object Hit task, the participant used both hands to hit balls that appeared at the far end of the screen and moved towards them, spanning different positions from the middle to the sides [34]. Participants encountered a total of 300 balls, and the speed of the balls increased progressively. The task was performed twice, but the analysis concentrated on the second attempt only to reduce any potential learning effect [35].

#### 2.5.2. Ball on Bar

In the Ball on Bar task, the participant was given a virtual bar to hold between their hands with a virtual ball positioned on it [36]. The goal was to successively reach four targets by positioning the virtual ball accurately on each target. The task includes three levels of difficulty, where the first level was the easiest and the third level was the most challenging, characterized by increased movement of the ball and a higher risk of it falling off the bar. The task was completed twice, with the analysis focusing on the second attempt to minimize learning effects [35].

#### 2.5.3. Visually Guided Reaching

In the Visually Guided Reaching task, the participant had to reach four targets that were distributed at a 10 cm radius from the initial target [11]. The targets were presented in a pseudorandom order. The aim was to reach the targets as quickly and precisely as possible. This task was performed with the LA arm first, and then with the MA arm.

### 2.6. Clinical Assessments

#### 2.6.1. Observation-Based Assessment of the Involvement of the MA Arm

In the Observation-based assessment of the involvement of the MA arm, the participant had to perform 7 different tasks (1—cleaning up the table; 2—making coffee; 3—setting the table for two; 4—pouring a glass of water from a pitcher; 5—cutting a piece of mastic; 6—folding two towels; 7—putting toothpaste on a toothbrush) while being video-recorded. The main goal of this assessment is to evaluate the integration of the MA arm during various bimanual tasks by scoring different aspects of the assisting hand’s performance, such as grasping and releasing (see [7] for the detailed list of criteria). Two clinicians have rated the relative involvement of the MA arm offline, as presented in [7], which has been shown to have a good reliability (τb = 0.84).

#### 2.6.2. Two-Arm Coordination Test

In the Two-Arm Coordination Test, the participants had to move an apparatus on a traced star as quickly and precisely as possible [37]. The task required bimanual coordination as both hands had to work together to successfully complete the task. Pushing or pulling on two handles allowed participants to perform up–down and right–left displacements. Participants performed the task four times in each direction (i.e., in a clockwise or counterclockwise direction), and the starting direction was randomized across participants. A one-minute practice session was conducted at the beginning of the testing to ensure that participants would be assessed on their motor performance and not their understanding of the task. The main variable for this test was the performance index, representing the multiplication of the time and the number of errors (i.e., number of times the stylus fell outside the drawing line) +1. Based on [10], an average of the third and fourth trials were used, as a learning plateau was demonstrated after the third trial. The average score for the clockwise and counterclockwise trials was calculated, as a Wilcoxon signed-rank test did not show a significant difference between the two directions. The Two-Arm Coordination Test was shown as a valid assessment to evaluate bimanual coordination in children having cerebral palsy [10].

#### 2.6.3. Jebsen–Taylor Hand Function Test

The Jebsen–Taylor Hand Function Test includes seven unilateral tasks (1—writing a sentence; 2—turning cards over; 3—picking up small objects; 4—simulating feeding; 5—stacking checkers; 6—lifting light objects, 7—lifting heavy objects) that were performed by the participants with their LA arm first and then their MA arm after [38]. The main variable was the total time required to complete all the tasks. The total time was converted into a z-score based on normative values provided in [38]. The Jebsen–Taylor Hand Function Test was shown as a reliable assessment to assess unilateral capacity in children having cerebral palsy [39].

#### 2.6.4. Box and Block Test

The Box and Block test required the participant to move blocks from one side of a wooden box to the other for 1 min. Participants were not allowed to throw blocks to the other side or to grab more than one block at a time. Participants had a 30 s practice period before starting the test to make sure they understood the instructions. The main variable was the number of blocks successfully sent to the other side of the box. The number of blocks was converted into a z-score based on the normative data provided in [40]. They performed the test twice, with the analysis focusing on the second attempt only as the Wilcoxon signed-rank test showed a significant difference between the first and the second attempt (*p* = 0.01). The Box and Block test was shown as a reliable assessment to assess unilateral capacity in children having cerebral palsy [41].

#### 2.6.5. Grip Strength

To measure the grip strength, a hand grip dynamometer was used (Jamar, Performance Health, Mississauga, ON, Canada). Participants were asked to grip the handle of the dynamometer as hard as they could, and the peak value of the attempt was recorded. Three attempts were conducted for each hand, and the mean value of these trials was used for analysis. The testing was performed in a random order between hands, with a 1 min break between each trial to avoid fatigue. In cases where there was a high discrepancy across trials, a fourth trial was conducted, and the two most similar values were then averaged. The mean of the peak values obtained was then converted to a z-score based on the normative values provided in the hand grip dynamometer user’s guide [42]. Reliability of the Jamar hand dynamometer was shown for the assessment of grip strength in individuals having cerebral palsy [43].

### 2.7. Statistical Analysis

Descriptive statistics (comprising mean and standard deviation (SD)) were calculated for sociodemographic variables and accelerometry metrics. One participant was excluded from the *Use Ratio* analyses as they were an outlier and the inclusion of their results would have led to an abnormal distribution of data. After this exclusion, normality testing showed that all data were normally distributed (see Figure S1 in the Supplementary Materials). Kruskal–Wallis tests were performed on accelerometry metrics (i.e., *Magnitude Ratio*, *BAUI*, *Use Ratio*, *Percentage of Bilateral Use*) to assess the difference in upper extremity use across levels of impairments (MACS). Stepwise, forward, and backward regression analyses were performed for accelerometry metrics according to two regression models: 1—the Robotic Assessment Model (comprising the following tasks: Object Hit, Ball on Bar, and Visually Guided Reaching); and 2—the Clinical Assessments Model (comprising the following assessments: Observation-based assessment of the involvement of the MA arm, Two-Arm Coordination Test, Jebsen–Taylor Hand Function Test, Box and Block test, and grip strength). The adjusted r-squared value (*r^2^*) for each model and accelerometry metric was reported to account for differences in the number of values entered in each model. Two models were employed to account for the important differences between robotic and clinical assessments (notably, the fact that clinical assessments have a strong focus on manual function while robotic assessments focus on proximal motor control). Our objective was to identify the most significant contributor for each assessment method. For all statistical analyses, the alpha threshold was set to 0.05.

## 3. Results

### 3.1. Sample Description

**Figure 3:**
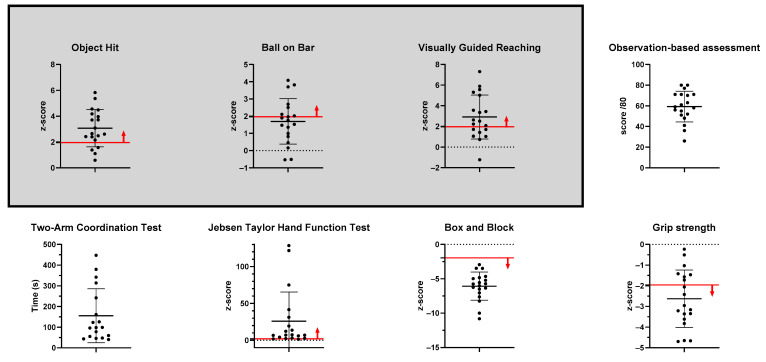
Histograms showing the distribution of results for each assessment. Robotic assessments are displayed within a grey square. The red line indicates the cutoff for normative data when available, with the direction of the arrow pointing toward the presence of impairments.

Nineteen participants were recruited for this study (see Table 2 for information on sociodemographic and clinical characteristics). Eighteen of the participants were able to complete all planned assessments, while one participant was unable to complete the Two-Arm Coordination Test due to poor grasping capacities (i.e., the participant was unable to hold the handles). One participant performed only one attempt for the Object Hit and the Ball on Bar tasks due to muscle fatigue and an increase in spasticity. The results of the first attempt were used for this participant as the Wilcoxon signed-rank test showed no effect of trial at the group level (a comparison between first and second attempts resulted in a *p*-value > 0.05). Table 3 and Figure 3 show the distribution of the results of motor assessments.

### 3.2. Description of Upper Extremity Use Using Accelerometry During Kitchen Tasks

**Figure 4:**
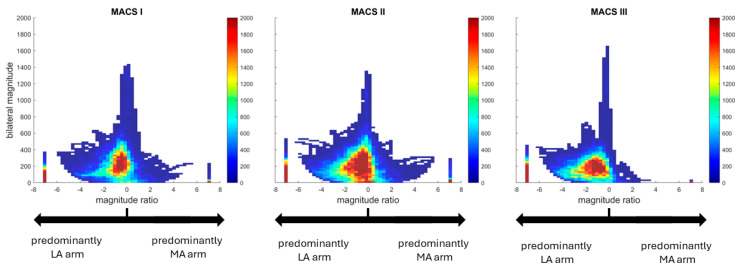
Distribution of upper limb use intensity for representative subjects across levels of impairment. The *x*-axis represents the *Magnitude Ratio*, i.e., the relative contribution of each upper extremity in terms of intensity. A negative value represents a larger use of the MA arm while a positive value represents a larger use of the LA arm. A value of 0 means that both arms are used equally. The column of data at −7 represents unilateral movement of the LA arm, and the column of data at 7 represents unilateral movements of the MA arm. The *y*-axis represents the intensity of bilateral use (*Bilateral Magnitude*). The color scale represents the frequency at which each combination happened, ranging from blue (less frequently) to red (more frequently). MACS = Manual Ability Classification Scale.

Figure 4 displays density plots that illustrate the distribution of the intensity of upper limb use intensity in three representative subjects, one for each of the MACS levels (see Figure S2 in the Supplementary Materials for the density plot for each of the 19 subjects). The *Bilateral Magnitude* (*y*-axis on the density plots) was similar across MACS levels ((mean ± SD), MACS I: 140.4 ± 29.7; MACS II: 147.9, ± 31.6; MACS III: 118.8 ± 65.4) which shows that the intensity of the combination of MA and LA arm movements did not change based on the severity of participants’ impairments, with the LA arm compensating any lack of MA use. The shift of the density plot toward the left (i.e., negative *Magnitude Ratio*) shows that the participants used their LA arm with a greater intensity during both bilateral and unilateral movements and that this asymmetry was more important among more severely impaired individuals. The column of data seen at a *Magnitude Ratio* of 7 showed that adults with a MACS level of III displayed almost no unilateral movements with their MA arm, while less impaired individuals still used their MA arm unilaterally, although to a lesser extent (i.e., the column at 7 is smaller than the column at −7).

Table 4 reports descriptive data for each accelerometry metric of interest and compares these metrics across MACS levels. The results show that the two metrics that focus on intensity (i.e., *Magnitude Ratio* and *BAUI*) are distinguished across MACS levels, but this is not the case for metrics that focus on duration of use, although a trend was observed for the *Use Ratio*, showing that participants with worse motor capacities tend to use their MA arm less.

### 3.3. Relationship Between Robotic and Clinical Assessments and Upper Limb Use in Real-Life Activities

Table 5 shows the results of the stepwise and forward regression analyses as they provided identical results. The results for the backward models are not presented for clarity but yielded identical results for the intensity-based metrics and similar results for the duration-based metrics. The presented models were chosen based on the results of the previous section (i.e., intensity-based metrics allowed us to distinguish across MACS levels). For the Robotic Assessment Model, the only variable entered in the model was the Ball on Bar test (a bilateral symmetric common goal task). For the Clinical Assessments Model, for most of the metrics (except for the *Magnitude Ratio*), the variable integrated in the model was the Observation-based assessment of the involvement of the MA arm (bilateral independent goal task). Interestingly, most of the assessments integrated in the models were bilateral tasks, regardless of whether the accelerometry metrics focused on global relative use or specifically on bilateral use.

## 4. Discussion

The first objective of this study was to describe relative MA arm use during an ecological task and test whether it differed across different levels of motor impairments (MACS I, II, and III) in adults with CP. The results indicate that metrics focusing on intensity, as opposed to duration of use of each arm, have a better capacity to discriminate across the different levels of manual impairment, as assessed using MACS. The second objective was to assess the relationship between relative use of the MA arm and motor functions, assessed with either robotic or clinical assessments. Among the metrics focusing on intensity, the *Magnitude Ratio* was the one being predicted by both robotic and clinical assessments (r^2^ of 0.43 and 0.82, respectively). However, the same robotic assessment, the Ball on Bar task, was identified as the best predictor of accelerometry metrics. For the clinical assessments, most of the accelerometry metrics were predicted by the Observation-based assessment of the involvement of the MA arm, while the *Magnitude Ratio* was predicted by the Box and Block test and the Jebsen–Taylor Hand Function Test. These results reaffirm the importance of assessing the use of both arms during ecological tasks. Indeed, it is well established that the majority of activities of daily living require the coordinated use of both arms [16,23,44,45], a concept known as bimanual coordination. The term “bilateral use”, as used in this article, refers to the use of the upper extremities regardless of whether they are used simultaneously or sequentially. This term was preferred to enhance clarity and encompasses both the coordination and use of the upper extremities as assessed in this study.

*Magnitude Ratio* and *Use Ratio* have been studied in various populations, allowing for a comparison with data from the literature. The range of the *Magnitude Ratio* (−0.69 to −1.63) observed in the present study clearly reveals asymmetry of the arm used compared with the one reported in healthy subjects (−0.06 to −0.16) [26,44,46] (a value of 0 indicated equal use of both arms). These assessments are quite well aligned, although they suggest a bit less asymmetry in our sample, with results being reported in the individual with stroke (i.e., −1.3 to −2.2) [26,47] and children with CP (i.e., −1.7) [48]. The same observations are seen in the *Use Ratio*, showing a clear asymmetry, even among participants classified as MACS I (i.e., 0.3 to 0.47) compared with reference values in healthy subjects (i.e., 0.71 to 1.00) [21,46,49,50] (*Use Ratio* = 1 represents equal use of both arms). Our results contribute additional insights into the bilateral use of upper extremities, encompassing both the time spent moving both arms simultaneously and the relative intensity of movements when this occurs. Specifically, our findings reveal that asymmetry of intensity between both arms during simultaneous movements (referred to as *BAUI*) also make it possible to discriminate across levels of manual impairments. Overall, the fact that only metrics that focused on intensity of use (*Magnitude Ratio* and *BAUI*) were able to discriminate between levels of manual impairments suggests that these metrics should be favored over those that focus on duration of arm use.

Despite the different ability of accelerometry metrics to discriminate across levels of manual impairments, they all showed a similar pattern of results for the regression models, in which Ball on Bar and the Observation-based assessment of the involvement of the MA arm were almost systematically identified as the best predictors. It is interesting to note that these two assessments focus on bimanual function, suggesting that impairments in bimanual function specifically interfere with the successful integration of the MA arm in an ecological task. Among the robotic assessments, two bilateral tasks were tested: a symmetric common goal task (Ball on Bar) and an asymmetric independent goals task (Object Hit). Although several components of these two tasks have been shown to be impaired in adults with CP [14], our results align with those of Decraene et al. [17], who demonstrated that the Ball on Bar task offers a more discriminative assessment than the Object Hit task when evaluating different aspects of bilateral functioning (i.e., spatiotemporal coupling and interlimb differences) in children with CP. On the one hand, the fact that the Observation-based assessment of the involvement of the MA arm was the best predictor of accelerometry is not surprising, as this assessment focuses specifically on the integration of the MA arm into bimanual activities, which is what is measured with accelerometry. On the other hand, this tool relies on observation, while many other clinical assessments offer a more quantitative approach and normative data. This finding is in line with the findings of a study reporting that the frequency of wrist movements measured with accelerometry was correlated to the observer’s report during the Assisting Hand Assessment test in children with CP (i.e., our Observation-based assessment of the involvement of the MA arm was developed based on the Assisting Hand Assessment test) [51]. Notably, the tasks that were assessed were mainly asymmetric with independent goals. While this might appear to contradict the results of the Robotic Assessment Model, important differences between the tasks need to be kept in mind. For example, the Object Hit task has much higher requirements for quick visuomotor processing compared with the everyday tasks (e.g., cooking or folding towels) performed in the corresponding clinical assessment. Another important difference is that, in the Observation-based assessment of the involvement of the MA arm, participants were instructed to use their arm as they typically would at home, allowing them to employ their preferred strategies. In contrast, the other clinical assessments provided specific instructions on how to perform the tasks, minimizing compensatory movements (e.g., during the Jebsen–Taylor Hand Function Test, participants were required to use a cylindrical grasp when lifting the can). This could explain the strong relationship between the Observation-based assessment of the involvement of the MA arm and the accelerometry metrics as they assess closely related concepts. When looking at the two common goal tasks, the Two-Arm Coordination Test requires the ability to correctly hold the handles of the apparatus, which was sometimes a limiting factor, while the Ball on Bar test required only movements of shoulders and elbows. Nevertheless, the results of both models support the importance of performing assessments that specifically focus on bimanual functions in individuals with CP.

### 4.1. Clinical Relavance

The accelerometry metrics indicate that, even among less severely impaired participants, adults with CP tend to underuse their MA arm despite having the capacity to use it. This tendency is even more pronounced in individuals with greater impairments. This is a significant observation, as most adults with CP do not receive treatment for their upper extremity impairments [52], further reinforcing the phenomenon of developmental disregard. This underscores the critical need for targeted interventions for adults with CP, who are often left on their own upon reaching adulthood. Our findings demonstrate the necessity of interventions focusing on arm use and particularly on the intensity of use. The small *Magnitude Ratio* and *BAUI* not only indicate that adults with CP use their MA hand less frequently but also reveal that it is primarily used to support the movement of their LA arm. These results highlight the need for a paradigm shift in CP care for adults. The fact that CP is considered a non-progressive disorder does not mean that nothing can be done to improve their functional abilities.

### 4.2. Study Limitations

This study has several limitations that should be acknowledged. First, the small number of participants included in the study reduced the generalizability of results. However, recruiting an evenly distributed sample across the three MACS levels helped mitigate the risk of bias. The small number of participants did not allow for analyses based on age and sex. This would have been an interesting consideration, as it is well established that motor capacity declines with age [53,54] and that there is a difference between the motor function of men and women [55]. Second, the lack of normative data for two of the clinical assessments used (i.e., the Observation-based assessment of the involvement of the MA arm and the Two-Arm Coordination Test) may have influenced the relationship with the accelerometry metrics. This issue reflects a longstanding challenge for adults with CP, as many assessments were originally developed for different populations or for children, leading to a scarcity of objective assessments of bilateral coordination tailored specifically to adults with CP. The robotic system used in this study did not provide an ecological environment to assess upper extremity functions as it is not representative of everyday life. Moreover, the position of the arm in the robotic device did not allow for measuring the pronation/ supination, which is important when performing activities of daily living. These limitations may have contributed to the weaker relationship found in the Robotic Assessment Model as the robotic device did not assess hand dexterity or postural control and removed the impact of gravity on upper extremities, limiting the impact of weakness. Fourthly, we collected accelerometry data for a specific set of tasks, including cooking, setting the table, eating, and drinking, which is not fully representative of the integration of the MA arm across the broader spectrum of activities of daily living. Our laboratory-based study allowed us to assess unilateral and bilateral functions in a controlled environment, serving as an essential first step. Further studies are needed to explore upper limb integration across a broader range of activities. Finally, we used a clinical measurement for grip strength that only considers the peak value of muscle contraction. This approach does not allow for an analysis of the dynamics of muscle contraction. Investigating this aspect would be an interesting avenue for future studies, as it is known that children with CP exhibit a different pattern of muscle activation compared with a control group [56].

## 5. Conclusions

In conclusion, the integration of robotic, clinical, and accelerometry assessments offers valuable insights into the factors contributing to upper extremity use in adults with CP. Our results support the use of intensity-based accelerometry metrics to measure MA arm use during daily activities and robotic or clinical assessments that focus on bimanual function to predict MA arm integration during ecological tasks, such as cooking or eating. More studies targeting the difference of bilateral task demands are required to better understand the mechanisms that explain developmental disregard as it leads to long-term disabilities [57]. Moreover, to address the limitations of the current study, further research should include a larger sample size, examine the impact of age on bilateral use, and gather accelerometry data outside of the laboratory.

## Figures and Tables

**Table 1:** Assessment details for each session.

Session 1 (<3 h)	Session 2 (~3 h)
Informed consent and initial clinical history interview (~30 min) Object Hit (~7 min) Ball on Bar (~8 min) Visually Guided Reaching of the MA and LA arm (~10 min) Two-Arm Coordination (~15 min) Jebsen–Taylor Hand Function Test of the MA and LA arm (~20 min) Box and Block test (~10 min) Grip strength (~8 min)	Observation-based assessment of the involvement of the MA arm (~30 min) Kitchen task (~1 h 30 min to 2 h 30 min)

Legend: Robotic assessments are in blue, clinical assessments are in green, and accelerometry assessment is in red. Three hours was the maximum duration of the session for people having more impairments and having more fatigue (i.e., they required more frequent and longer breaks).

**Table 2:** Sociodemographic and clinical characteristics of the participants.

Total Number of Participants	19
Age (years)	34.3 ± 11.5
Sex (female (F), male (M))	F = 58% M = 42%
More affected side (right (R), left (L)) *	R = 63% L = 37%
Handedness (right (R), left (L)) *	R= 42% L = 58%
Percentage of participants for each level of the Manual Ability Classification System	I = 37% II = 31.5% III = 31.5%

* Note that one participant was right-handed but their more affected arm was their right arm.

**Table 3:** Mean and 95% confidence interval (z-score) by motor assessment.

		Mean [95%—Confidence Interval]
Robotic Assessments	Object Hit	3.1 [2.37–3.76]
	Ball on Bar	1.7 [1.66–3.9]
	Visually Guided Reaching	2.9 [1.9–3.9]
Clinical Assessments	Observation-based assessment of the involvement of the MA arm	59.3 [52.1–66.4]
	Two-Arm Coordination	155.5 [90.6–220.4]
	Jebsen–Taylor Hand Function Test	25.9 [7.0–44.9]
	Box and Block test	−6.1 [−10.8–−2.9]
	Grip strength	−2.6 [−3.3–−1.96]

Legend: Grey cells represent robotic assessments.

**Table 4:** Mean (±standard deviation) of each accelerometry metric and their comparison across levels of impairment.

		MACS I	MACS II	MACS III	Total	*p*-Value
Intensity	*Magnitude Ratio*	**−0.69 ± 0.46**	**−1.02 ± 0.28**	**−1.63 ± 0.75**	**−1.09 ± 0.64**	**0.047**
	*Bilateral Arm Use Index*	**0.71 ± 0.18**	**0.52 ± 0.10**	**0.47 ± 0.14**	**0.58 ± 0.18**	**0.03**
Duration	*Use Ratio*	0.47 ± 0.09	0.37 ± 0.10	0.30 ± 0.16	0.38 ± 0.13	0.15
	*Percentage of Bilateral Use*	26.1 ± 13.5	24.2 ± 6.9	16.2 ± 14.0	22.4 ± 12.1	0.42

Legend: The left column indicates whether the metric focused on intensity or duration of use. Cells in grey correspond to metrics that focus on global use, while white cells correspond to metrics that focus on bilateral use. The significance level for the Kruskal–Wallis tests was set at *p* ≤ 0.05. Significant results appear in bold; MACS = Manual Ability Classification Scale.

**Table 5:** Results of the stepwise and forward regression analysis for each accelerometry metric, using either the Robotic Assessment Model or the Clinical Assessment Model.

		Robotic Assessment Model	Clinical Assessment Model
**Intensity**	*Magnitude Ratio*	Ball on Bar (r^2^ = 0.43, *p* = 0.001)	1- Box and Block test (r^2^ = 0.72, *p* < 0.001) 2- Box and Block test and Jebsen–Taylor Hand and Function (r^2^ = 0.82, *p* < 0.001)
	*Bilateral Arm Use Index*	Ball on Bar (r^2^ = 0.23, *p* = 0.02)	Observation-based assessment of the involvement of the MA arm (r^2^ = 0.60, *p* < 0.001)
**Duration**	*Use Ratio*	Ball on Bar (r^2^ = 0.27, *p* = 0.02)	Observation-based assessment of the involvement of the MA arm (r^2^ = 0.64, *p* < 0.001)
	*Percentage of Bilateral Use*	Ball on Bar (r^2^ = 0.25, *p* = 0.02)	Observation-based assessment of the involvement of the MA arm (r^2^ = 0.43, *p* = 0.002)

Legend: The Robotic Assessment Model comprised the following tasks: Object Hit, Ball on Bar, and Visually Guided Reaching. The Clinical Assessment Model comprised the following tests: Observation-based assessment of the involvement of the MA arm, Two-Arm Coordination Test, Jebsen–Taylor Hand Function Test, Box and Block test, and the grip strength test. Grey cells represent the metric focusing on the relative use of the upper extremity.

## Data Availability

The raw data supporting the conclusions of this article will be made available by the authors without undue reservation.

## Supplementary Materials

The following supporting information can be downloaded at: https://www.mdpi.com/article/10.3390/s25030660/s1, Figure S1: Distribution of data for the four accelerometry metrics. Mean and standard deviation values are displayed; Figure S2: Distribution of upper limb use intensity for each subject.
