# Supplementary material for: Exploring the Link Between Motor Functions and the Relative Use of the More Affected Arm in Adults with Cerebral Palsy
Source: Sensors (Basel). 2025 Jan 23;25(3):660. doi: 10.3390/s25030660 (PMC11819957; doi:10.3390/s25030660)
Supplement: Supplementary file 1 [file sensors-25-00660-s001.zip › sensors-3394925-supplementary.pdf]

sensors

Supplementary Materials

1.1 Supplementary Figures

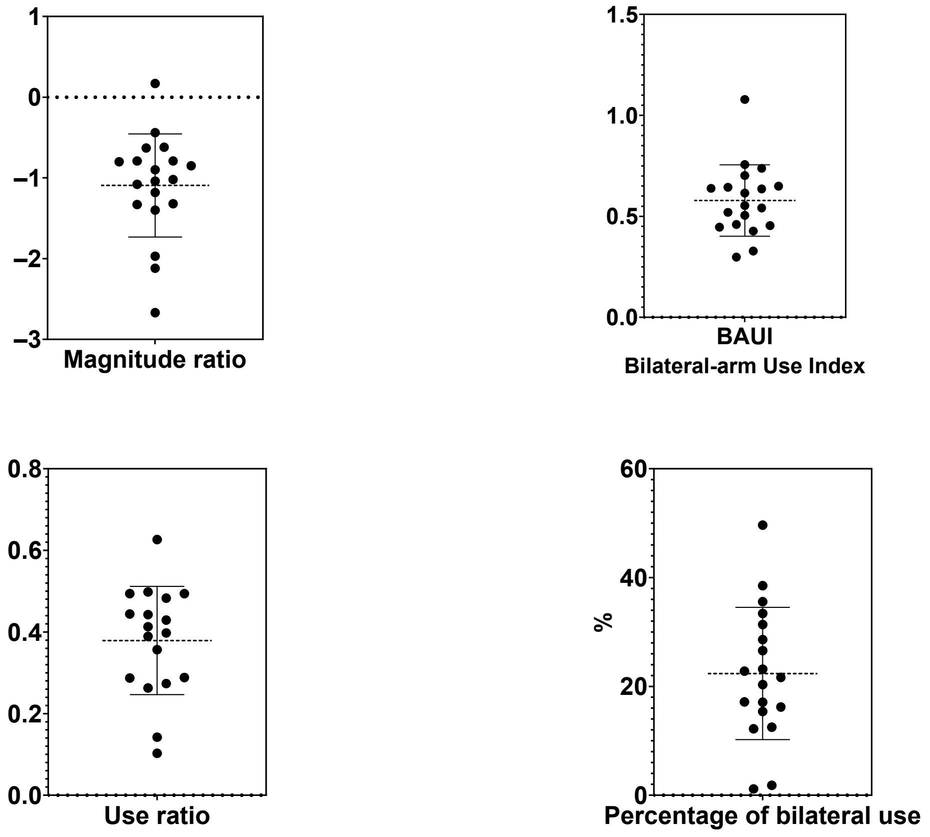

Figure S1. Distribution of data for the four accelerometry metrics. Mean and standard deviation values are displayed.

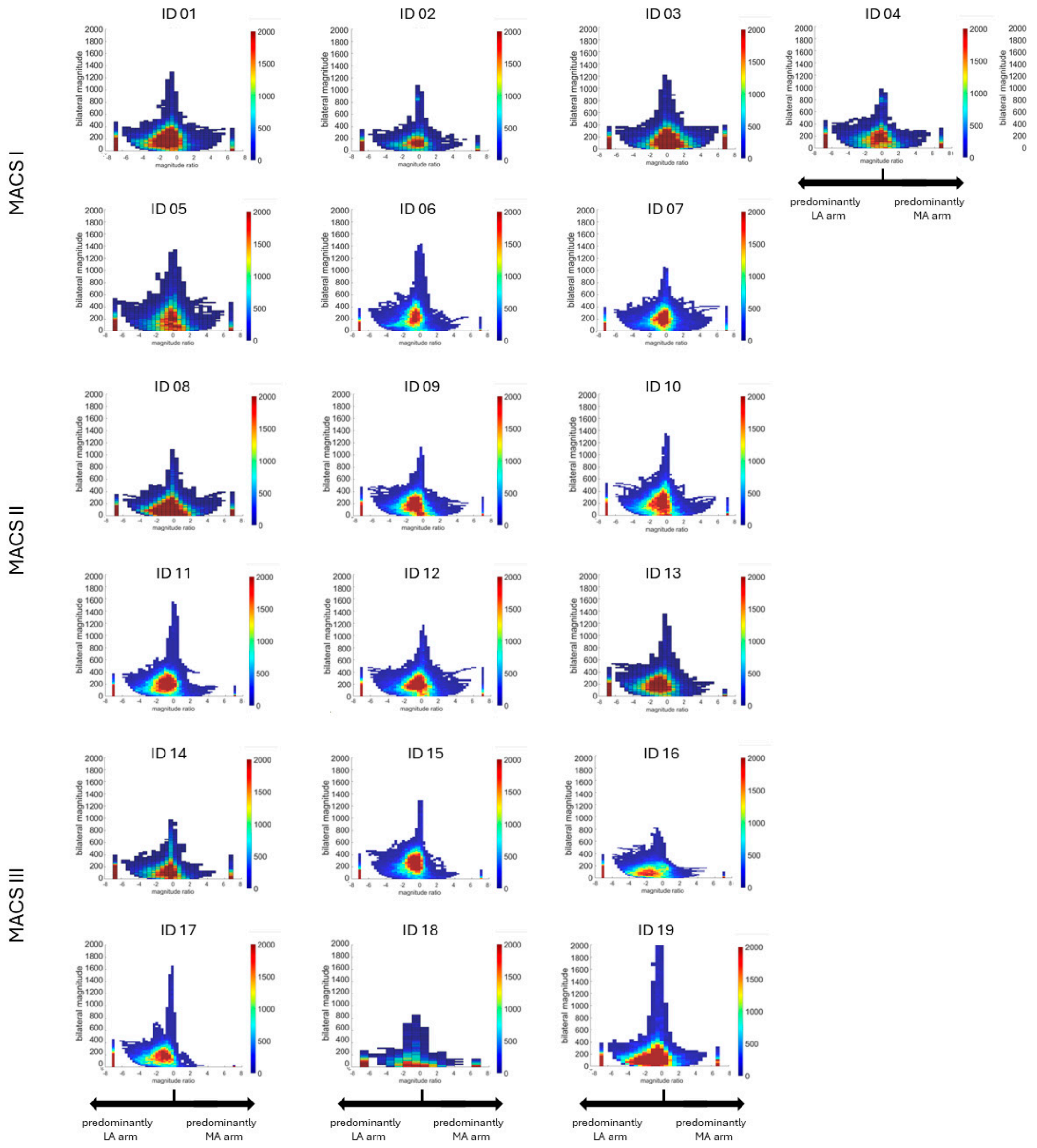

Figure S2. Distribution of upper limb use intensity for each subject. The x-axis represents the magnitude ratio, i.e. the relative contribution of each upper extremity in terms of intensity. The column of data at -7 represents unilateral movement of the LA arm, and the column of data at 7 represents unilateral movements of the MA arm. The y-axis represents the intensity of bilateral utilization. The color on the graphic represents the frequency at which each combination happened. The scale goes from blue (less frequently) to red (more frequently).
